# Earthworm-Derived Pore-Forming Toxin Lysenin and Screening of Its Inhibitors

**DOI:** 10.3390/toxins5081392

**Published:** 2013-08-08

**Authors:** Neelanun Sukumwang, Kazuo Umezawa

**Affiliations:** 1 Department of Applied Chemistry, Faculty of Science and Technology, Keio University, Kanagawa 223-8522, Japan; E-Mail: neela_nun@hotmail.com; 2 Department of Molecular Target Screening, School of Medicine, Aichi Medical University Nagakute, Aichi 481-1195, Japan

**Keywords:** Lysenin, *Eisenia foetida*, hemolysis, pore formation, sphingomyelin binding, *Dalbergia latifolia*, all*-E*-lutein, tyrosylproline anhydride

## Abstract

Lysenin is a pore-forming toxin from the coelomic fluid of earthworm *Eisenia foetida*. This protein specifically binds to sphingomyelin and induces erythrocyte lysis. Lysenin consists of 297 amino acids with a molecular weight of 41 kDa. We screened for cellular signal transduction inhibitors of low molecular weight from microorganisms and plants. The purpose of the screening was to study the mechanism of diseases using the obtained inhibitors and to develop new chemotherapeutic agents acting in the new mechanism. Therefore, our aim was to screen for inhibitors of Lysenin-induced hemolysis from plant extracts and microbial culture filtrates. As a result, we isolated all-*E*-lutein from an extract of *Dalbergia latifolia* leaves. All-*E*-lutein is likely to inhibit the process of Lysenin-membrane binding and/or oligomer formation rather than pore formation. Additionally, we isolated tyrosylproline anhydride from the culture filtrate of *Streptomyces* as an inhibitor of Lysenin-induced hemolysis.

## 1. Introduction

Pore-forming toxins are mainly produced as soluble proteins. The pore-forming toxins typically transform from soluble, monomeric proteins to oligomers that form transmembrane channels. The monomeric form of the toxin is bound to the membrane where its oligomerization is induced. This process generates stable target membrane insertion, resulting in the transmembrane pore formation. This effect depends on the concentrations of toxins and the distance from the site of production [1].

Many pore-forming toxins have been discovered in various organisms, including prokaryotes and eukaryotes [2]. Bacterial pore-forming toxins include a cholesterol-dependent toxin (streptolysin O) from *Streptococcus pyogenes*, cytolysins from *Escherichia coli*, aerolysin from *Aeromonas hydrophila*, a toxin from *Staphylococcus aureus*, and hemolysin from *Clostridium septicum* and *Vibrio cholerae* [3]. Pore-forming toxins are also observed in animals, such as that in the sea anemone named *Metridium senile* [4]. Cytolysin is a toxin from *Actinia equine* [5], and hydralysin is secreted from green hydra named *Chlorohydra viridissima* [6]. Considering plant sources, enterolobin is a toxin generated in *Enterolobium contortisiliquum* [7].

A number of pore-forming toxins have been found to interact with sphingomyelin. For example, sticholysin I and II secreted from *Stichodactyla helianthus*, cytolysin from *Vibrio cholerae*, pleurotolysin from the *Pleurotus ostreatus* mushroom, Lysenin from the earthworm *Eisenia foetida*, all prefer sphingomyelin-containing membrane to form pores [8]. Lysenin is a hemolytic protein derived from the coelomic fluid of the *Eisenia foetida* earthworm. This protein is known to bind sphingomyelin specifically and induce lysis of erythrocytes. However, the precise mechanisms of Lysenin-induced hemolysis remain to be elucidated. 

We also screened for cellular signal transduction inhibitors of low molecular weight from microorganisms and plants. Microorganisms and plants produce many bioactive metabolites of low molecular weight with unique structures. The purpose of the screening was to study the mechanism of diseases using the obtained inhibitors and to develop new chemotherapeutic agents acting in the new mechanism. Previously, we isolated protein-tyrosine kinase inhibitors, protein-tyrosine phosphatase inhibitors, anti-Ras compounds, and NF-κB inhibitors. These inhibitors all ameliorated disease models in animals. Screening for inhibitors of Lysenin-induced hemolysis would be one of the possible approaches to better understand the mechanism of Lysenin’s action. Inhibitors of Lysenin-induced hemolysis may be useful as anti-inflammatory agents. Moreover, Lysenin should activate innate immunity by disturbing the target membrane structure, especially if it is a sphingomyelin-binding protein. Thus, Lysenin and the newly found inhibitors should be useful in studying the mechanism of inflammatory diseases, and additionally, inhibitors of Lysenin-induced hemolysis may be useful as anti-inflammatory agents. Therefore, we have aimed at screening inhibitors of Lysenin-induced hemolysis from plant extracts and microbial culture filtrates.

## 2. Structure of Lysenin and Induction of Hemolysis

**Figure 1:**
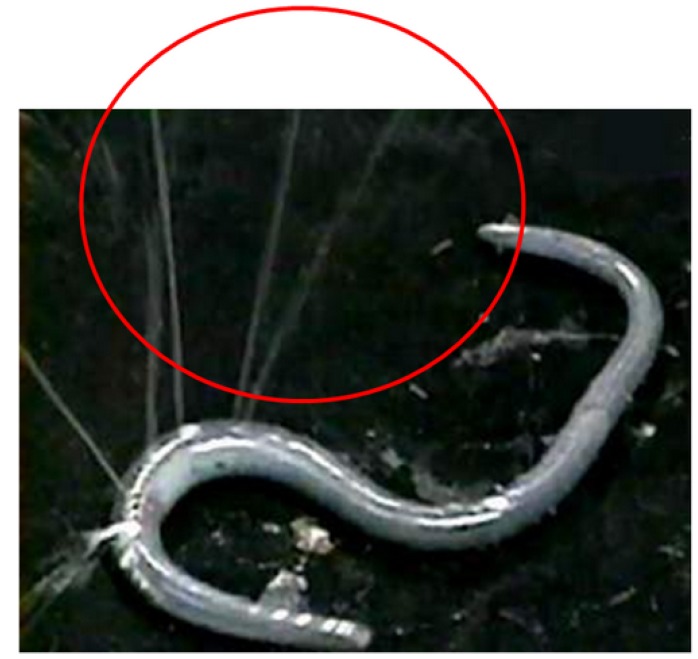
Earthworm *Eisenia foetida* ejecting coelomic fluid.

Lysenin was cloned in 1997 for determining the protein that induces contraction of rat vascular smooth muscle from the coelomic fluid of *Eisenia foetida* [9]. The protein was then reported as a sphingomyelin-binding protein [10]. Injection of the coelomic fluid supernatant into the vein of rats, mice and quails induces death, and the active principle is also Lysenin [11]. This earthworm is categorized in a subclass of Oligochaeta in the phylum of Annelida [12]. *Eisenia foetida* ejects its coelomic fluid when attacked or stimulated, as shown in Figure 1. Lysenin is a pore-forming toxin existing in the coelomic fluid of the earthworm *Eisenia foetida*. This protein specifically binds to sphingomyelin and induces erythrocyte lysis. Lysenin consists of 297 amino acids with a molecular weight of 41 kDa. Lysenin induces hemolysis and is toxic to vertebrate spermatozoa, amphibian larvae, and cultured mammalian cells, such as normal spleen cells, colon cancer cells, and breast cancer cells, and also amphibian larvae [10,13,14,15]. Opper *et al.* demonstrated by flow cytometry and immunocytochemistry that the highest amount of lysenin is expressed in the cell called chloragocytes, which is one subgroup of earthworm immune cells also called coelomocyte [16]. Recently, the structure of Lysenin was studied by crystallographic analysis, and Colibus *et al.* have suggested it shares a common ancestry with other pore-forming proteins from a diverse set of eukaryotes and prokaryotes [17].

In the coelomic fluid, Lysenin consists of a family of proteins together with Lysenin-related protein1 and Lysenin-related protein2 [9]. The sequence of amino acids of Lysenin is more homologous to that of Lysenin-related protein2 than that of Lysenin-related protein1.

Lysenin can induce hemolysis, and the Lysenin-induced hemolysis occurs in a temperature-dependent and dose-dependent manner as evidenced by previous studies [10,18]. The amount of sphingomyelin in the membrane also affects hemolysis induction by Lysenin [10]. Lysenin contains six tryptophan residues and five of them are conserved in Lysenin-related protein1 and Lysenin-related protein2. Recent studies have shown that conserved tryptophan could be important in the recognition of sphingomyelin and hemolytic activity [19].

**Figure 2:**
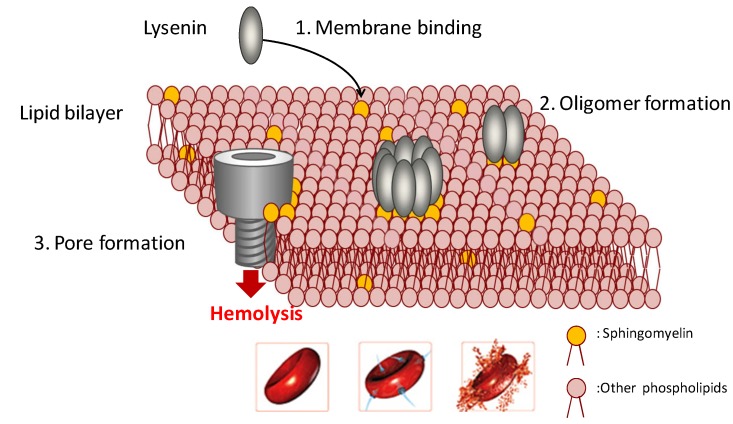
Pore formation by Lysenin.

The interaction of Lysenin to erythrocyte membranes containing sphingomyelin occurs in three stages, in which the initial stage is attachment of Lysenin to sphingomyelin of the target membrane; the second stage, the formation of oligomers that induce an increase in membrane permeability; and the final stage, the formation of the mature pores on the membrane inducing hemolysis (Figure 2). Pore formation depends on environment temperature. Cell lysis occurs more easily at 37 °C compared to that at 4 °C. The membrane pore size formed by Lysenin is estimated at around 3 nm [18].

Recently, many researchers from various fields have attempted to clarify the mechanisms of hemolysis induced by Lysenin. A study by Ishitsuka and Kobayashi demonstrated that cholesterol and sphingomyelin/Lysenin ratio influenced oligomerization [20]. Binding of Lysenin to sphingomyelin was inhibited by the presence of glycolipid, thus hemolysis decreased [21]. An electrophysiological investigation showed that Lysenin molecules formed voltage-dependent ion-channels in artificial lipid bilayer membranes. Moreover, some of the lipid components in the membrane bilayer influenced the channel activity [22]. It was suggested that an α-helix portion of Lysenin would be a possible membrane inserting fragment of the protein [23]. 

## 3. Isolation of All*-E*-Lutein as an Inhibitor of Lysenin-Induced Hemolysis

For the evaluation of hemolytic activity, we incubated 3 × 10^7^ cells/mL packed sheep erythrocytes with 50 ng/mL Lysenin at 37 °C for 30 min, with or without screening sample. A total of 1030 samples of plant extracts and microbial culture filtrates were screened for inhibitors of Lysenin-induced hemolysis. 

**Figure 3:**
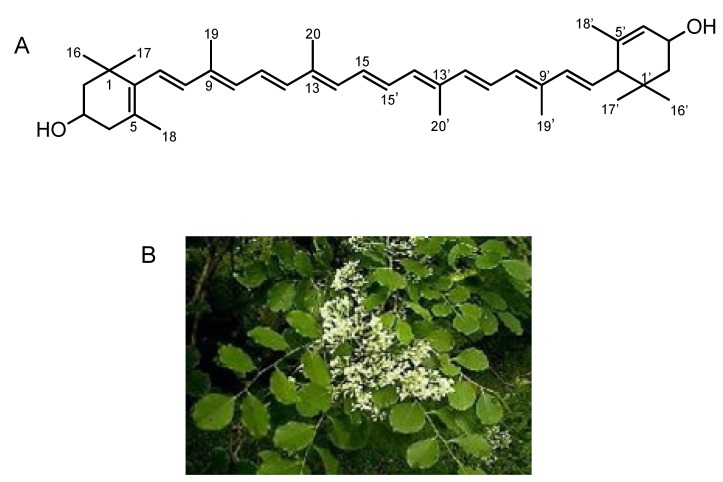
(**A**) All-*E*-Lutein; (**B**) All-*E*-lutein-producing plant *Dalbergia latifolia*. It belongs to the family of *Fabaceae* (*Leguminosae*), and is commonly called East Indian rosewood or black rosewood

As a result, we found a botanical product of *Dalbergia latifolia* (Indian rosewood) as a possible inhibitor. A methanolic extract of *Dalbergia latifolia* leaves showed strong inhibitory activity toward Lysenin-induced hemolysis [24]. The active substance was isolated from the raw materials as an orange solid by using solvent extraction and chromatographic separation procedures. The UV-Vis spectra showed absorption maxima in MeOH at 270, 335, 420(sh), 447 and 475 nm. The positive ion mode of MS-ESI showed the base peak at *m*/*z* 551.4, corresponding to the [M + H − H_2_O]^+^, and HR-ESI-MS (pos) showed *m/z* 568.4299 (M^+^) (calcd. for C_40_ H_56_O_2_, 568.4275). These NMR data were almost identical with those of the known carotenoid, all-*E*-lutein [25,26]. Finally, the active compound was identified as all*-E*-lutein (Figure 3) in good agreement with previously published data [25,26,27].

## 4. Lysenin-Induced Hemolysis and the Effect of All-E-Lutein

**Figure 4:**
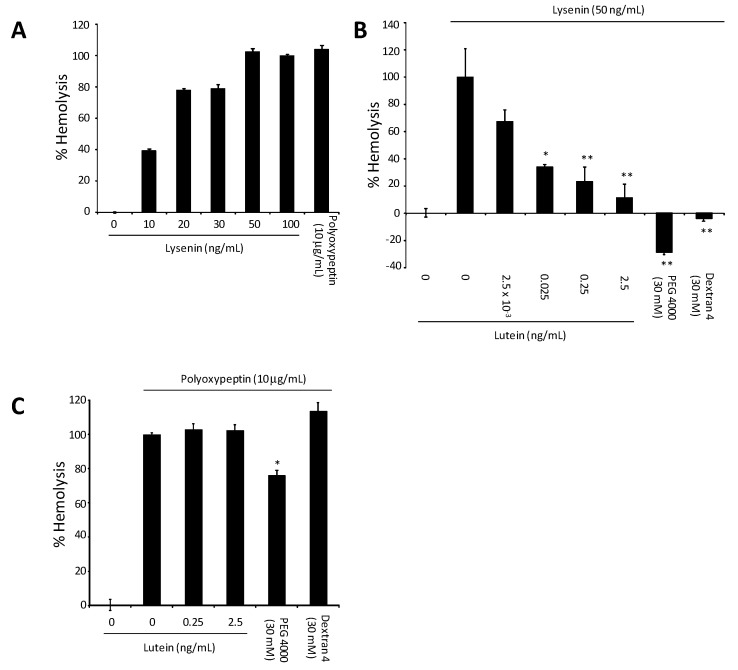
(**A**) Induction of hemolysis by Lysenin in sheep red blood cells; (**B**) Inhibition of Lysenin-induced hemolysis by all-*E*-lutein. PEG 4000 and dextran 4 are known inhibitors of hemolysis; (**C**) All-*E*-lutein does not inhibit polyoxypeptin A-induced hemolysis. The Data are mean ± S.D. of experiment performed in triplicate. (* *p* < 0.05, ** *p* < 0.01).

Lysenin induced hemolysis at 10–100 ng/mL, as shown in Figure 4A. The hemolysis result was compared to that of polyoxypeptin A (as positive control). As shown in Figure 4B, all*-E*-lutein inhibited the Lysenin-induced hemolysis dose dependently at 0.025–2.5 ng/mL. PEG 4000 and dextran 4 are known to be hemolysis inhibitors [18], and they perfectly inhibited the Lysenin-induced hemolysis at 30 mM. Inhibition of Lysenin-induced hemolysis by all-*E*-lutein is likely to be specific, since it did not inhibit the polyoxypeptin A-induced hemolysis (Figure 4C). Although dextran 4 did not inhibit the polyoxypeptin A-induced hemolysis, PEG 4000 partially inhibited (about 20%) the polyoxypeptin A-induced hemolysis at 30 mM.

Lysenin binds and forms oligomers on membranes of erythrocytes, and these steps are temperature-independent processes that can occur at 4 °C. In contrast, the pore formation is induced only at 37 °C. First, the effect of all-*E-*lutein on the membrane-binding process and oligomer formation was evaluated. Sheep erythrocytes in the presence of all*-E-*lutein were incubated with Lysenin 50 ng/mL at 4 °C. Then the cells were incubated without all*-E-*lutein. Hemolysis was obviously inhibited by all*-E-*lutein, possibly by suppressing the Lysenin-membrane binding and/or oligomer formation processes. Because PEG 4000 and dextran 4 inhibit hemolysis in a pore size-dependent manner [18], these inhibitors did not inhibit the Lysenin-membrane binding and/or oligomer formation processes. Next, the effect of all*-E-*lutein on Lysenin-induced pore formation was evaluated. Sheep erythrocytes were preincubated with Lysenin without all*-E-*lutein, resuspended, and then treated with all*-E-*lutein in the presence of Lysenin at 37 °C. The all*-E-*lutein did not inhibit the hemolysis in this procedure. PEG 4000 and dextran 4 inhibited pore formation. This result indicates that the pore-formation process mediated by Lysenin was not affected by all*-E-*lutein. 

*Dalbergia latifolia* is a plant in the family of *Fabaceae*. It is a premium-quality timber species known as “Indian rosewood”, which is widely used for manufacturing furniture and ornamental products. This plant is also known to be useful against termites [28], as well as a medicinal plant for the treatment of diarrhea, indigestion, and spermatorrhoea [29]. *Dalbergia latifolia* leaves have been used in folk medicine. The whole of *Dalbergia latifolia* can be used to treat against infection by parasitic worms (helminths) [30], and a methanolic extract of the leaves also has an inhibitory effect on nitric oxide production in lipopolysaccharide-stimulated RAW 264.7 cells [31]. In addition to all*-E*-lutein, *Dalbergia latifolia* is known to be a source of latifolin, 4-methoxydalbergione, obtusaquinol, and dalbergiphenol, which are used for treatment against termites and fungi [28]. Thus, our results suggest all-*E*-lutein to be another active compound derived from *Dalbergia latifolia* leaves, this one being an inhibitor of Lysenin-induced hemolysis.

## 5. Isolation of Tyrosylproline Anhydride as an Inhibitor of Lysenin-Induced Hemolysis

**Figure 5:**
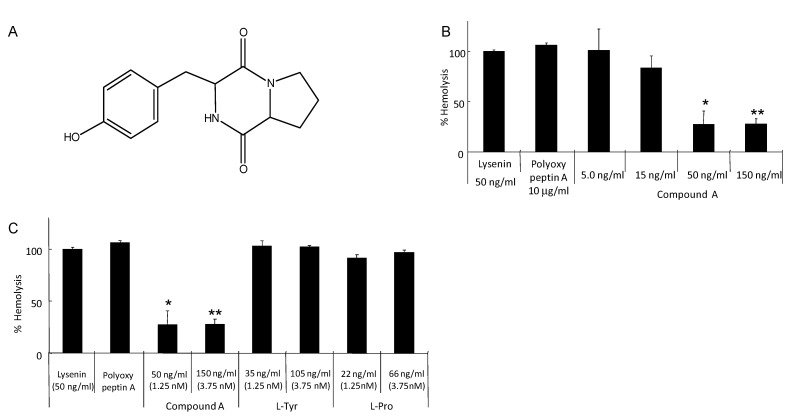
(**A**) Tyrosylproline anhydride; (**B**) Inhibition of Lysenin-induced hemolysis by tyrosylproline anhydride; (**C**) Neither tyrosine nor proline inhibits Lysenin-induced hemolysis. The Data are mean ± S.D. of experiment performed in triplicate. (* *p* < 0.05, ** *p* < 0.01)

We continued screening Lysenin-induced hemolysis inhibitors from the culture filtrate of microorganisms employing the same assay system. After testing about 550 samples, we found a positive effect in the culture filtrate from the *Streptomyces* sample. The positive sample of 750 mL culture filtrate was extracted with butanol and dried, resulting in 680 mg of yellow residue. Then, the residue was applied to a silica gel column, LH20 column, and HPLC to give 3 mg of pure compound A. The active principle (compound A) was isolated with several chromatographs, and the structure was determined by mass spectra, NMR spectra including proton, carbon-13, DEPT, COSY, HMQC, and HMBC. Finally, compound A was determined to be tyrosylproline anhydride, as shown in Figure 5A. The absolute structure of compound A remains to be determined. This compound inhibited the hemolysis at 15–50 ng/mL, as shown in Figure 5B. Each fragment, tyrosine or proline, showed no effect (Figure 5C). L-Tyr-L-Pro anhydride was named as maculosin, and it is produced by *Alternaria alternata*, spotted knapweed [32]. It shows phytotoxic and antibiotic activities.

Thus, we have found that tyrosylproline anhydride from *Streptomyces* inhibits Lysenin-induced hemolysis. This may be a new activity of maculosin.

## 6. Conclusions

Lysenin is a pore-forming protein derived from the coelomic fluid of the *Eisenia foetida* earthworm. This protein can effectively induce hemolysis in sheep red blood cells. We have screened low molecular weight inhibitors of Lysenin-induced hemolysis using sheep red blood cells from plants and microorganisms. As a result, we isolated all*-E*-lutein from the extract of *Dalbergia latifolia* leaves. All-*E*-lutein likely inhibits the process of Lysenin-membrane binding and/or oligomer formation, rather than pore formation. We have isolated trosylproline anhydride from the culture filtrate of *Streptomyces* as an inhibitor of Lysenin-induced hemolysis. Since pore-forming melittin and Staphylococcal delta hemolysin [33] are inflammatory agents, Lysenin is likely to be an inflammatory agent. These inhibitors may be useful in understanding the mechanism of Lysenin action, and furthermore, they may be useful as anti-inflammatory agents.
